# High-resolution, quantitative 3D PET image reconstruction for the Siemens hybrid 3T MR/BrainPET scanner using the PET reconstruction software toolkit (PRESTO)

**DOI:** 10.1186/2197-7364-1-S1-A51

**Published:** 2014-07-29

**Authors:** Juergen Scheins, Christoph Weirich, Liliana Caldeira, Philipp Lohmann, Elena Rota Kops, Lutz Tellmann, Michaela Gaens, Hans Herzog, Uwe Pietrzyk, N Jon Shah

**Affiliations:** Institute of Neuroscience and Medicine-4, Forschungszentrum Juelich, Kragujevac, Germany

**Figure 1:**
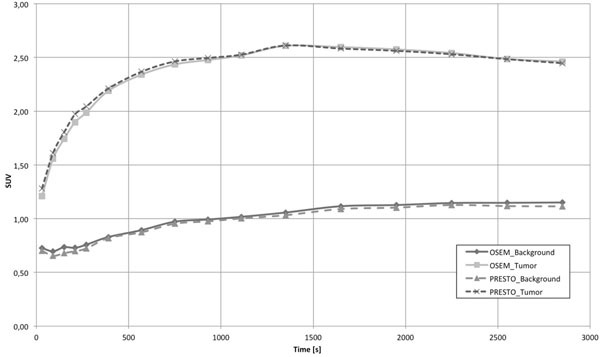
Comparison of TAC of a dynamic FET human brain tumor measurement.

**Figure 2:**
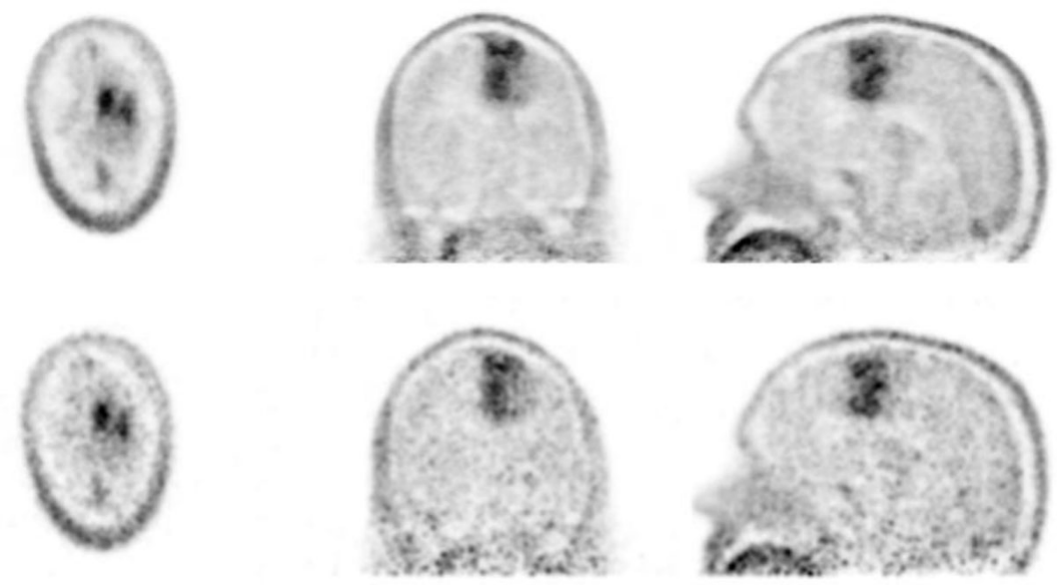
Image quality of a FET brain tumor measurement; PRESTO (top) vendors’ reconstruction (bottom).

The Siemens 3T MR-BrainPET scanner allows us to simultaneously acquire high-resolution MR and PET images thus giving a strong asset for studies of the human brain. Meanwhile, the system is routinely used for MR-PET studies with a variety of radiotracers, e.g. ^18^F-FDG, ^18^F-FET, ^11^C-Raclopride, ^11^C-Flumazenil, ^15^O-Water. Based on the vendors’ sinogram-based reconstruction, quantitative dynamic images are obtained. However, this reconstruction uses compressed data in terms of span (axial) and mash (transaxial). Avoiding such data reduction strategies is desirable to improve the image quality. In this context, the PET Reconstruction Software Toolkit (PRESTO) provides better image quality in terms of resolution and noise at the expense of increased computational effort. For the first time, an accurate quantification with PRESTO has been achieved by integrating all mandatory data corrections. All data corrections are calculated for LORs individually and passed to the OP-OSEM implementation of PRESTO. The corrections comprise: component-based normalisation, template-based attenuation correction, variance-reduced random correction, scatter correction based on Single Scatter Simulation, dead time/pile up correction, decay correction and system calibration. In this way, the reconstructed images provide calibrated time-activity (TA) values (Bq/cc). Comparisons between TA curves (TAC) from the sinogram-based reconstruction and PRESTO show reproducible values within a few percent for all available tracers. Exemplarily, Figure 1 compares the brain tumor dynamics for a scan with FET. No significant deviations are observed in the TACs. However, the better SNR becomes evident for PRESTO (Figure 2). Consequently, the hybrid 3T MR-BrainPET has emerged as an excellent tool for a wide spectrum of PET studies of the human brain due to the continuous improvements, which have successfully addressed the issues of quantification, optimising image quality and workflow.

